# Effect of glycerol concentration on levels of toxicants emissions from water-pipe tobacco smoking (WTS)

**DOI:** 10.1186/s12889-023-16740-2

**Published:** 2023-09-25

**Authors:** Salwa Almomen, Mobarak Aldossari, Yousef Khaleel, Mishal Altamimi, Ohoud Alharbi, Abdulelah Alsuwaydani, Malak Almutairi, Sarah Alyousef, Radwan Hafiz, Faris Alshomer, Amani S. Alqahtani

**Affiliations:** 1 Saudi Food and Drug Authority (SFDA), 4904 Northern Ring Branch Rd, Riyadh, Saudi Arabia; 2 Saudi Food and Drug Authority, 3904 Northern Ring Road, Hittin-Riyadh, 1351307148 Saudi Arabia; 3 Eurofins-Ajal, 14813 Al Anwar St, Riyadh, Saudi Arabia

**Keywords:** Waterpipe smoking, Waterpipe tobacco, Tobacco regulation

## Abstract

Glycerol, flavorings and sweeteners constitute approximately 70% of water-pipe tobacco smoking (WTS) mixtures. Tobacco mixture combustion produces smoke toxins (e.g. carbonyl compounds), of which the type and amount are highly dependable on tobacco mixture formula. While glycerol in tobacco mixture contribute to enhanced smoking experience, its’ combustion produces toxicants such as acrolein. According to WHO, there are no approved international upper limits regulations on WTS ingredients. This study aims to assess toxicant emission levels corresponding to increasing glycerol concentration in WTS mixtures, which may aid in developing tobacco regulations towards harm reduction.

**Methods**

Laboratory experimental study. Using laboratory water-pipe smoking machine, levels of toxicant emissions in the smoke from WTS mixture samples containing varying glycerol concentrations were measured using High-performance Liquid Chromatography (HPLC). Smoke from 5 consecutive smoking cycles with 35 puffs each (ISO 22486 standard) was led through a trapping system as described in the Cooperation Centre for Scientific Research Relative to Tobacco (CORESTA) recommended method No. 74 (Determination of selected carbonyls in mainstream cigarette smoke by HPLC). Trapped carbonyls were then analysed by HPLC with a DAD detector.

**Results**

Acrolein emission is associated with glycerol addition in WTS mixture indicated by lab-made samples throughout all glycerol concentrations (10%, 20%, 40% and 60%), and brand samples with glycerol concentrations 10% to 20%. However, brand samples showed no increase in acrolein emission corresponding to the increase in glycerol concentrations from 20% to 60%.

**Conclusion**

The effect of glycerol addition in waterpipe tobacco on acrolein emission varies between products. Tobacco fillers, additives and contents quality and other factors may affect toxicant emission levels. Therefore, regulatory recommendations towards defining upper limits of content concentrations require further investigations regarding potential confounders in acrolein emissions and health effects of market-available glycerol concentrations in waterpipe tobacco smoking.

## Introduction

Waterpipe tobacco smoking (WTS) “also known as Hookah, Shisha, Arghile and Maassal” has been known to populations around the world since at least four centuries ago [1, 2]. Since the introduction of tobacco flavoring in early 1990s, WTS has become popular worldwide among male and female of all adulthood ages [3–8]. WTS is particularly prevalent in Eastern Mediterranean and Eastern European countries [3–6]. In 2011, a survey by (Maziak et al.) described a prevalence of 6–36% adolescent smokers in Middle Eastern countries [9]. Likewise, WTS in western countries has been increasingly reported to be gaining popularity over the last decade. In 2015, a study among students in the US described WTS prevalence of 33.8% in male and 28.4% in female [7, 8].

The rise in WTS use worldwide appears to be largely attributable to a well-perceived misconception by smokers that WTS is ‘less harmful and addictive’ than cigarettes due to its’ more intermittent use pattern and other smoking mechanism-related misbeliefs [10]. Alarmingly, WTS health effects were demonstrated to be equivalent to at least 100 cigarettes when WTS session lasts for 20–80 min (World Health Organization, 2013) [11]. Evidence by some studies indicated WTS association with cardiac and pulmonary disease [12, 13].

Tobacco constitutes around 30% of the mixture used in WTS, while the remaining 70% consists of flavorings, glycerol and sweeteners, hence the name Maassel which means ‘honeyed’ [14]. The combustion of such ingredients produces smoke particulates including nicotine, nitrosamines and carbonyl compounds. Many of those emissions, such as formaldehydes and acetaldehydes, are known toxins and carcinogens. The type and amount of toxic emissions generated in the vapor smokers exhale are highly dependable on the formulation of the smoked mixture [15]. Glycerol is added to WTS mixtures mainly for its water-retaining properties providing stability and quality to the mixture, flavor enhancement and the cloud-like characteristic of the smoke [16, 17]. Glycerol is not considered a toxic compound; however, its combustion generates known toxic and carcinogenic emissions, specifically acetaldehyde, benzaldehyde, acrolein, and acetone [18]. Bang G. Ooi et. al. and other studies demonstrated that the levels of toxic emissions are significantly higher with increased glycerol concentrations in the smoked mixture [18, 19].

Despite evident health concerns of WTS, studies examining waterpipe tobacco constituents’ emissions are still scarce. To this date, there are no regulations of WTS products composition in the US [12, 13, 20]. According to World Health Organization (WHO), there is a lack in the policy and regulations concerning the specifications of WTS products’ ingredients [2]. This results in the often exemption of those products from tobacco control policies especially in the developing countries [21, 22]. According to Saudi Food and Drug Authority (SFDA), the range of varying glycerol concentrations observed in market products oppose a public health concern in terms of toxicant emissions and safety. To our knowledge, the literature lacks evidence regarding acceptable glycerol upper limit concentrations in WTS. Therefore, investigating glycerol concentrations effects on toxicant emissions is important in WTS regulation development. In this study, we aim to measure levels of toxicant emissions from WTS mixture samples with different glycerol concentrations and, possibly, identify acceptable glycerol concentration range in WTS.

## Objectives

1. To measure the levels of toxicant emissions in smoke produced from WTS mixture samples with different glycerol concentrations.
2. To compare measured toxicant emission levels from WTS mixtures prepared by the experimental lab with their equivalent samples prepared by popular WTS companies (brands) in Saudi market.
3. To possibly identify acceptable glycerol concentration range in WTS based on the assessment of corresponding toxicant emissions measurements.

## Methodology

### Study design

Laboratory experimental study. Using laboratory water-pipe smoking machine, levels of toxicant emissions in the smoke from WTS mixture samples containing varying glycerol concentrations were measured using High-performance Liquid Chromatography (HPLC).

### Experimental plan

#### Experimental equipment

**Fig. 1:**
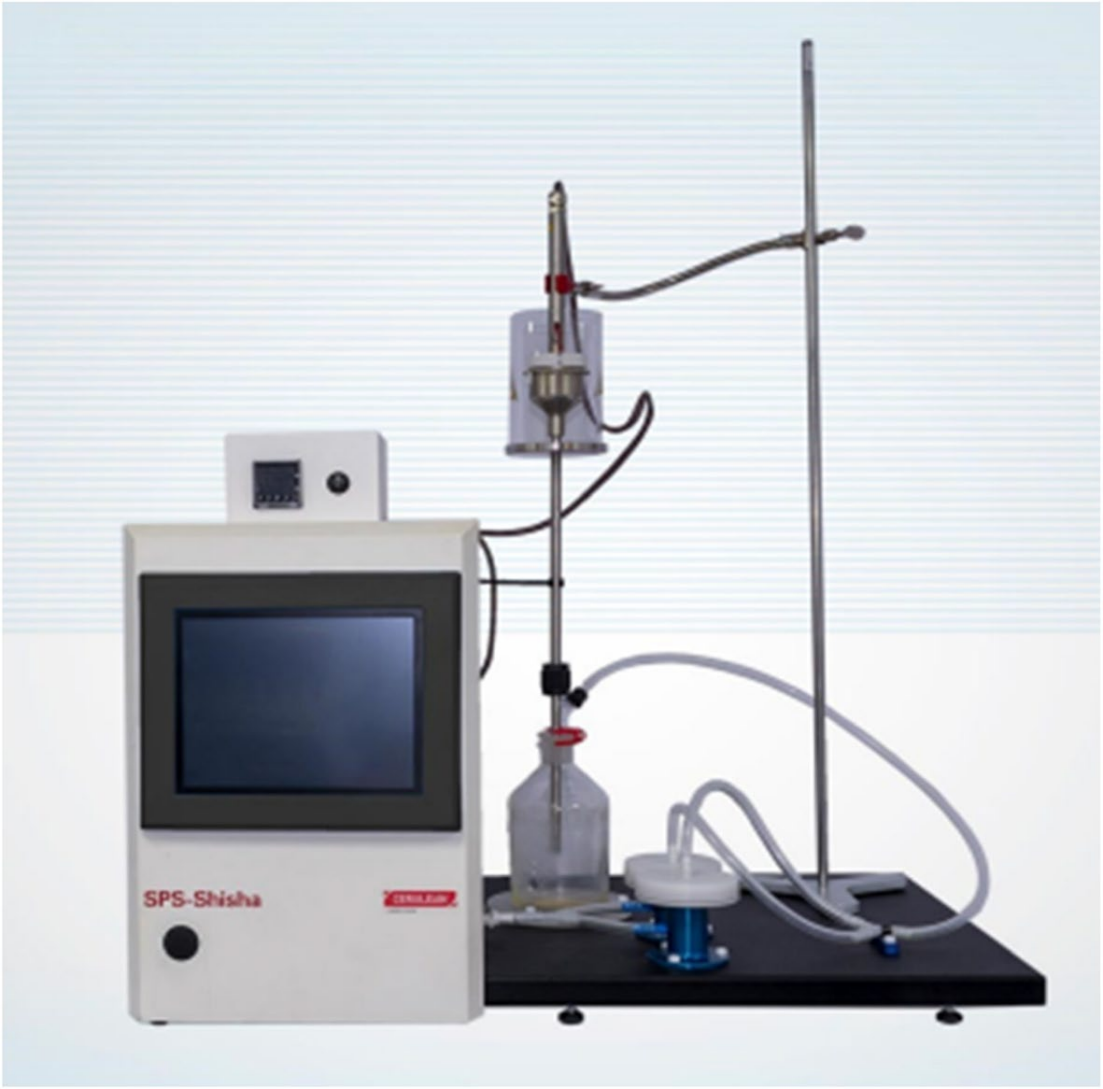
Laboratory water-pipe equipment (SPS SHISHA—Specialist shisha pipe smoking machine) complying with ISO 22486:2019 and used according to method ISO/TS 22487:2019

Laboratory water-pipe smoking machine (i.e hookah smoking simulator), of which water-pipe equipment principles are complying with the International Organization for Standardization (ISO) 22486:2019 and used according to method ISO/Water pipe tobacco smoking (ISO/TS) 22487:2019 [23, 24], see (Fig. 1).

#### Tobacco mixture preparation

A total of 12 WTS mixture samples were prepared for this study. Three manufacturers (lab, Company-1 and Company-2) prepared 4 WTS mixture samples each. Samples are comprised of:

- 2 basic compounds (tobacco and apple aroma flavors) with fixed concentrations.
- Sugar with concentration dependent on a ratio to glycerol concentration.
- Glycerol with concentrations varying in each of the 4 WTs mixture samples. The range of glycerol concentration for the 4 WTS mixture samples was determined as; a minimum point (10%) below the concentration range observed in market samples (20 – 35%), a maximum point (60%) above market sample range, and 2 average points (20%, 40%) in our samples range (10 – 60%).

In the study, WTS mixture samples are named (Lab-made, Company-1 and Company-2). Additionally, two control samples were prepared with only glycerol and no glycerol to serve as reference for specific toxicant emission association with glycerol. Table 1 illustrates preparation of all samples with specified glycerol and other basic compounds concentrations, as explained above. Company-1 and Company-2 samples were received in September and October 2021. The analysis duration was from beginning of November 2021 to end of December 2021. The amount of samples collected was 0.5 kg from each sample.

- Sample #1: constitutes 10% glycerol; the minimum point below the concentration range observed in market samples (20 – 35%).
- Sample #4: constitutes 60% glycerol; the maximum point above market sample range.
- Samples #2 and #3: constitute 20% and 40% glycerol, respectively; 2 average points in our samples range (10 – 60%).
- Control samples: these samples were prepared as reference/control to confirm toxicant emission specifically related to glycerol. Glycerol and glucose addition in WTs tobacco mixture is in the form of a complex compound with ratios of both glycerol and sugar that complement each other. Meaning, the higher the ratio of glycerol, the lower the ratio of sugar. The role of the 2 control samples in this study (one containing 65% glycerol, and another containing 0% glycerol) is to distinguish the effect of sugar alone on the emissions (using control samples containing 0% glycerol), and the effect of glycerol alone on the emissions (using control samples containing 65% glycerol). This demonstrate whether a specific emission such as acrolein, is attributed mostly to either glycerol or sugar, or to both of the compounds.

**Table 1:** Composition concentrations of WTS mixture samples named lab-made, company 1 and company 2

**Sample Specification**					
**Samples (lab-made, Company-1, Company-2)**	**Glycerol**	**Sugar**	**Tobacco**	**Flavor (apple aroma)**	**Total**
**1**	**10%**	**55%**	**30%**	**5%**	**100%**
**2**	**20%**	**45%**	**30%**	**5%**	**100%**
**3**	**40%**	**25%**	**30%**	**5%**	**100%**
**4**	**60%**	**5%**	**30%**	**5%**	**100%**

#### Lab-made mixture preparation

##### Sample contents

200 g ground raw tobacco material, 300 g distilled honey or sugar, flavor (apple aroma), glycerol. Details are shown in Table 1.

##### Preparation steps

1. A flat layer of ground raw tobacco material is spread in glass and plastic containers, ensuring an even surface level throughout the layer of tobacco. The layer is preferred to be of a low thickness so that it may be spread on the largest area possible.
2. The choice of flavor is sprayed onto the tobacco evenly and thoroughly with continuous mixing to ensure the delivery of flavor to all parts of the tobacco material.
3. Distilled honey or sugar is added to the tobacco and flavor mixture with constant mixing to ensure the delivery of distilled sugar to all parts of the mixture. Then, containers with tobacco mixture are sealed for 24 h.
4. After 24 h post tobacco-flavor-distilled sugar mixture preparation. Containers are unsealed to gently break down the mixture thickened mass into looser material.
5. Glycerol is added with continuous mixing to ensure delivery to all parts of the mixture.
6. Finally, thorough mixing is performed daily for 7 days to achieve high level of mixture homogeneity.
7. At this point, Lab-made sample is fully prepared and maybe used for water-pipe tobacco smoking.

#### Measured toxicant emissions in smoke

Nicotine, nitrosamines and carbonyl compounds were each analyzed in a separate experiment for all WTS mixture samples. All experiments were repeated (conducted twice) for all samples to obtain average readings for measured toxicants.

#### Smoke generation

A) Protocol: Each WTS mixture sample was smoked in the lab water-pipe smoking machine. A fixed mixture weight of 10 g for all samples was loaded in the machine. One smoking cycle consists of 5 × 35 puffs (i.e. 5 loads of sample) according to ISO 22486 [23].
B) Procedure: Chemical analysis was performed for smoke generated by a water-pipe smoking machine according to ISO 22486:2019 [23]. Although charcoal is typically used for water pipe smoking, an electrical heater at a temperature of 280°C was used according the ISO method. This was decided in order to eliminate the unpredictable influence of different types of charcoal on the measurement results.
  1. A sample of 10 g of WTP mixture was burnt for each test. Smoke was generated from 5 consecutive smoking cycles comprised of 35 puffs each (ISO 22486) [23].
  2. Next, the smoke was led through a trapping system as described in the CORESTA recommended method No.74 (Determination of selected carbonyls in mainstream cigarette smoke by HPLC) [25].
  3. Finally, trapped carbonyls were analyzed by HPLC with a Diode-Array Detection (DAD) detector.
  4. All results obtained from the analyses of carbonyl compounds were reported and documented in individual certificates for each sample in the Eurofins eLIMS system.

#### Chemical analysis

##### Carbonyl quantification

The determination of carbonyl compounds is carried out according to ISO 21160:2018 method (Cigarettes - Determination of selected carbonyls in the mainstream smoke of cigarettes - Method using high performance liquid chromatography) which is based on CRM No. 74. The smoking of waterpipe tobacco was according to ISO 21160:2018 (Cigarettes with a BKC Shisha Smoker); 5 runs with 35 draws and capturing of aldehydes by reaction to the corresponding hydrazone with 2,4-dinitrophenylhydrazine, Coresta-recommended method No. 74. (5 runs yield 5 individual samples). Heating temperature was set to 280 ° C. The electro heater (temperature set on 280 ° C) was laid on the tobacco. Five minutes later, the smoking cycles were started.

#### Quality control and assurance

The limits of quantification of carbonyls are as shown in Table 2.

**Table 2:** Limits of quantification (LOQ) for the analysis of carbonyl compounds

**Compound**	**LOQ (µg/ 35 puffs)**
Formaldehyde > 6	> 6
Acetaldehyde > 15	> 15
Acroleine > 12	> 12
Propionaldehyde > 12	> 12
Crotonaldehyde > 12	> 12
2-Butanone > 18	> 18
Butyraldehyde > 35	> 35

#### Laboratories participating in the study

1. Smoking of tobacco samples in the laboratory waterpipe instrument, analysis of carbonyl compounds and capturing of nitrosamines on filters: ASL Analytic Service Laboratory, Hamburg, Germany [26].
  This is an independent ISO 17025 accredited analytical laboratory (DAKKS number D-PL-19429-01-00) and an officially approved tobacco-testing laboratory within the European Union. ASL provides state-of-the-art analytical services related to tobacco and e-cigarettes for customers worldwide. ASL belongs to the Hauni group, the biggest producer –worldwide- of machinery for cigarette production and laboratory equipment (Borgwaldt) for cigarette analysis. This lab performs accredited tobacco analyses under ISO 17025 [26].
2. Analysis of the glycerol content in the tobacco samples: Eurofins Food & Feed Testing, Sweden, Lidköping [27]. This laboratory is accredited under ISO 17025 to analyze tobacco related nitrosamines and the content of additives like glycerol in tobacco [27].

### Data analysis

Average measurements of readings from two experiments (smoking sessions) for each sample were calculated. Linear regression was used to assess the correlation between glycerol content of WTS mixture samples (independent variable) and acrolein emissions in the produced smoke from those samples (dependent variable).

## Results

### Emission of total carbonyl compounds in the tobacco smoke

**Fig. 2:**
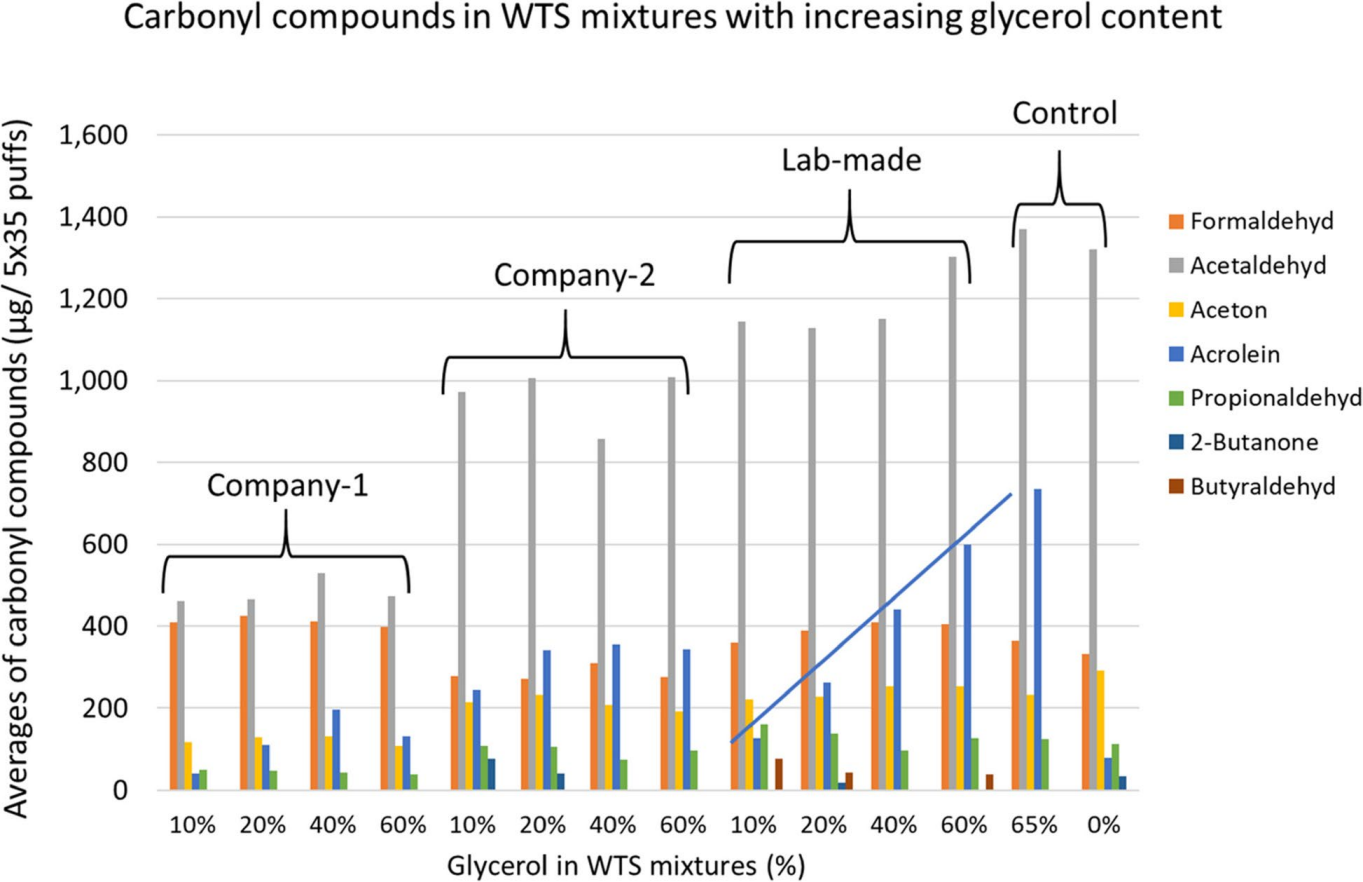
Emission of carbonyl compounds from the different tobacco samples with varying glycerol content. Average from 2 parallel smoking experiments per sample

Figure 2 shows the total emission of the different carbonyl compounds over the 5 smoking cycles with 35 puffs each. In all samples, there is no correlation between the glycerol content of the tobacco and the amounts of carbonyl compounds emitted during smoking, other than acrolein.

### Correlation between glycerol content and acrolein emission

In this study, control samples demonstrate that acrolein emission is attributed almost entirely to glycerol addition as acrolein emission was produced in very low concentrations from the control samples containing 0% glycerol, compared to its produced concentrations from control samples containing 65% glycerol which was very high (Fig. 2).

**Fig. 3:**
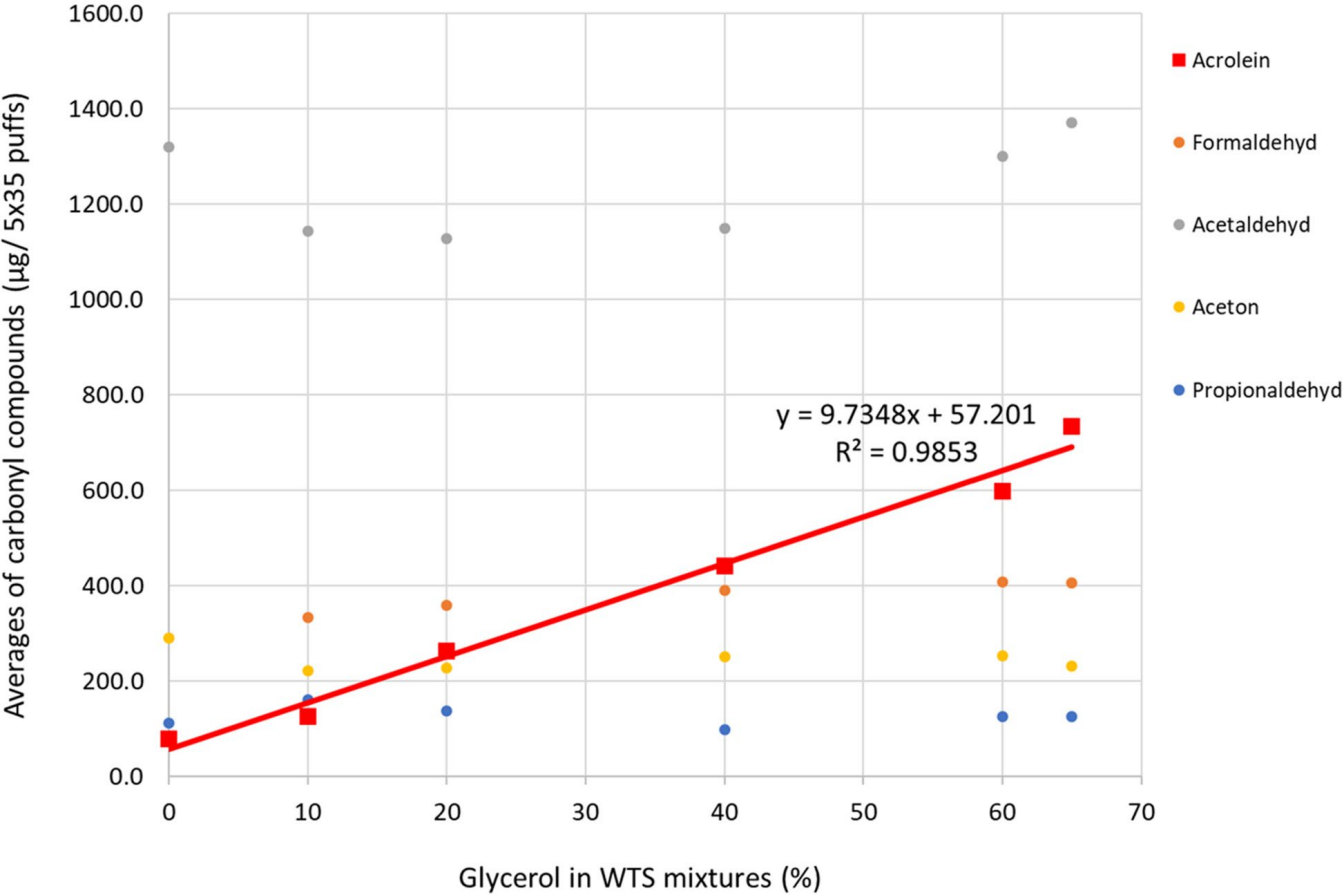
Regression between glycerol content of lab-made samples (including control samples) and acrolein emission, average from 2 parallel smoking experiments for each sample

In lab-made samples, the acrolein content in the smoke increases with the glycerol concentration of the tobacco in a direct correlation and a linear regression model can be applied (Fig. 3), in which the glycerol content is the independent and acrolein the dependent variable. The regression follows the formula y = 9.7348 x + 57.201 and the coefficient of determination is *R*^2^ = 0.9853. There is a significant correlation between glycerol addition and acrolein emission in lab-made sample (Fig. 3).

**Fig. 4:**
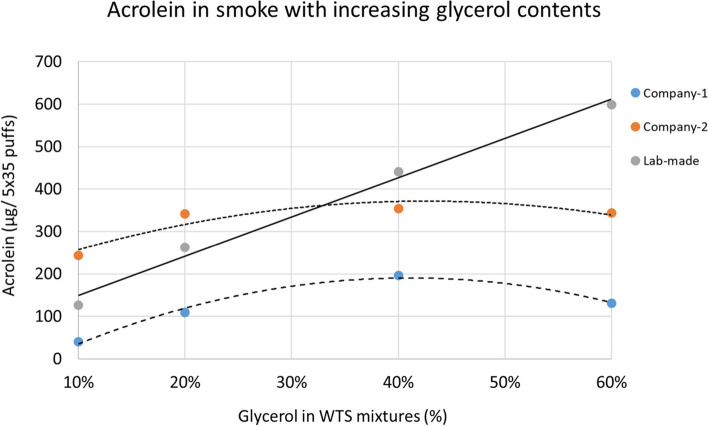
Acrolein formation in smoke from 5 × 35 puffs of commercial Company-1, Company-2 and lab-made WTS mixture samples with increasing glycerol contents

In contrast to lab-made WTS mixture samples, there is no correlation between the glycerol content, especially for concentrations above 20%, and acrolein emission in the commercial Company 1 and Company 2 samples (Fig. 4).

*P*-value for glycerol/acrolein with glycerol as the predictor variable is 0.00315181, which is far below 0.05 and we can conclude that glycerol concentration as a predictor for acrolein is statistically significant, in contrast to the couple glycerol/formaldehyde with a *p*-value of 0.673287041.

### Comparison of emission of other carbonyl compounds between companies and lab-made WTS mixture samples

**Fig. 5:**
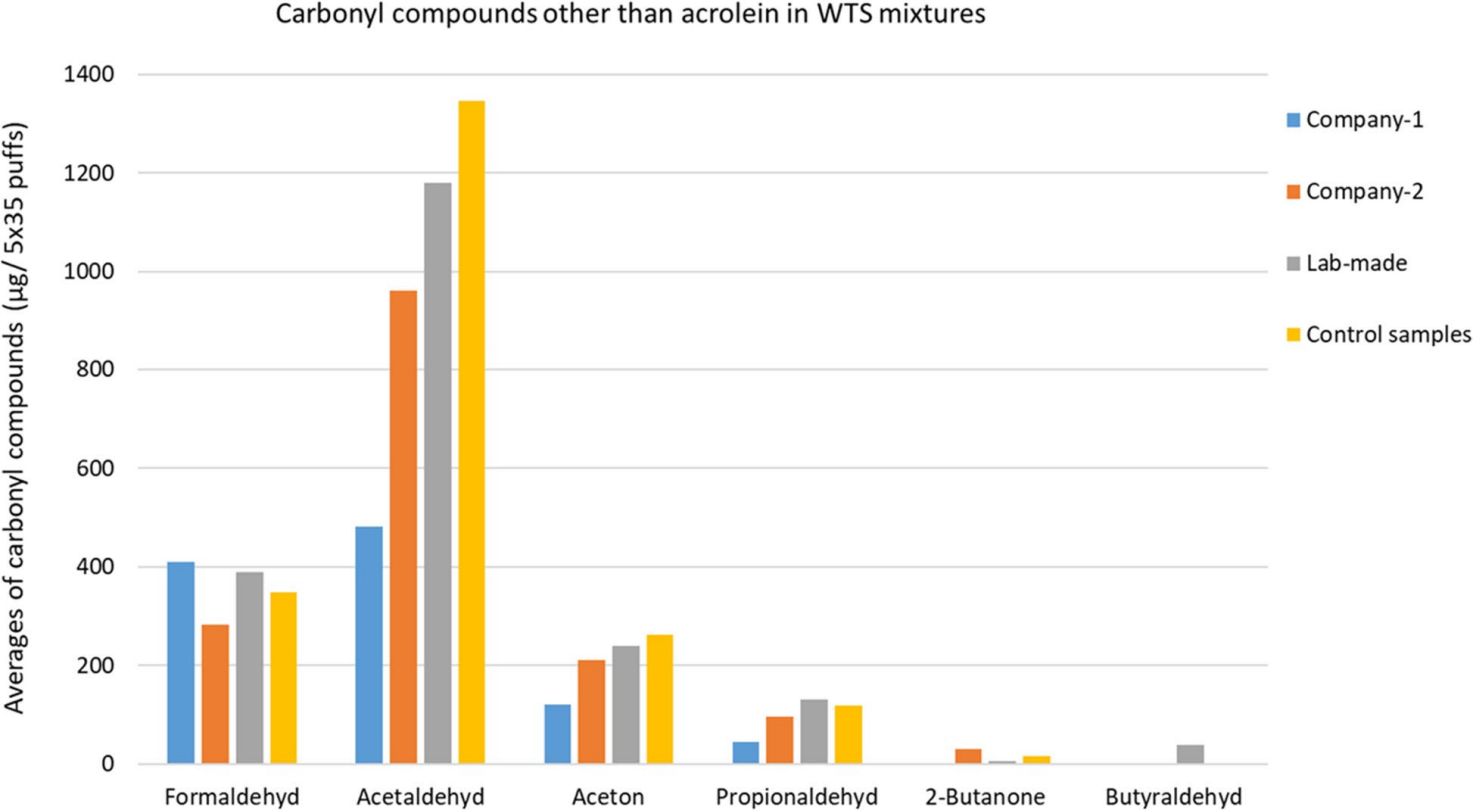
Comparison of carbonyl compounds emission between WTS mixture samples

Interestingly, the average emission levels of acetaldehyde, acetone and propionaldehyde from all samples are significantly higher in Company-2 and lab-made samples in comparison to Company-1 sample. Furthermore, no emission of 2-butanone was observed for the Company-1 samples, but occurred during burning of the Company-2 and lab-made samples (Fig. 5). Butyraldehyde was only emitted in concentrations above the limit of quantification by burning of the lab-made samples. The emission of formaldehyde was significantly higher from the Company-1 and lab made samples than from Company-2 samples.

## Discussion

### Acrolein association with glycerol

Of all toxicant emissions measured in this study, acrolein was found to increase in a directly proportional manner with glycerol concentration increase in lab-made WTS mixtures, and from 10% to 20% glycerol concentration increase in brand samples.

To our knowledge, evidence in the literature regarding glycerol incorporation in tobacco and its effect on acrolein emissions is still growing. However, the association between acrolein emission and glycerol addition has been described in the literature by several studies. A recent study in 2019, described glycerol influence on toxicant emission. They indicated that glycerol increase from 0% to 80% in E-liquids lead to acrolein emission increase by 28-fold [18]. Another showed an increase in acrolein production from 56 µg to 67–69 µg acrolein/cigarette, when glycerol concentration was increased from 1–5 wt% to 10–15 wt%, retrospectively [19]. In WTS products, glycerol concentrations are known to reach up to 64 wt%, presenting a potential health and/or air quality hazard from acrolein exposure [19].

### Acrolein emission in lab-made samples vs. commercial brand samples

As shown in (Fig. 4), Company-2 exhibits no increase in acrolein emission resulting from 20%-60% glycerol samples as emissions remain in the range of 200–300 µg/ 5 × 35 puffs. Likewise, Company-1 samples show no increase in acrolein emission as it reaches to 200 µg/ 5 × 35 puffs from 40% glycerol samples, then drops down to 100 µg/ 5 × 35 puffs from 60% glycerol samples.

Despite the overall increase in acrolein emissions from increasing glycerol concentrations up to 80% in E-liquids described by Bang G. Ooi et al., there was an observed slowdown in acrolein emission increase corresponding to glycerol concentration from 20% to 50% [18]. Similarly, our study showed a plateau in acrolein emission corresponding to this glycerol concentration range (20–60%).

It has been described in the literature that WTS mixture contents -beside glycerol- such as tobacco and propylene glycol contribute to acrolein emission as well [18, 28, 29]. Evidence from other studies indicated that acrolein is a by-product generating during a smoking session from the burning of tobacco as well as additives [19, 30]. Moreover, toxic aldehydes including acrolein are reported to be yielding from sugars as well [31]. A study demonstrated an emission increase from 118 µg to 215 µg acrolein/cigarette in produced smoke when 16% sucrose is added to cigarettes [32]. Furthermore, additives such as activate carbon may cause acrolein retention and filtration from emitted smoke. As well, more selective retention of acrolein was observed when using absorbents and resins with surface amino acids [33]. Evidently, glycerol is not a sole player in acrolein emission from water-pipe tobacco smoking. This may explain the varying levels of acrolein emission corresponding to different WTS mixture samples with equal glycerol concentrations. In other words, differences in acrolein emission levels may be expected when produced from the combustion of unidentical WTS mixtures in terms of additives and quality/grade of compounds. In this study samples are made using the same formula for basic ingredients, but were prepared by two different market brands as well as an experimental lab providing a generic product. The composition of the WTS provided by companies contain the same basic compound concentrations as specified in the study (Table 1), yet resembles additives or other ingredients that are otherwise present in companys’ market products. This is important to keep commercial samples in the study as similar as possible to products available in the market. Commercial samples were validated by Eurofins-Ajal labs to confirm the compliance of companies to specifications of basic compound concentration requested by the study. However, ingredients additional to the specified basic compounds by the study that may be present in commercial samples are not declared by the companies as they are not obligated to disclose ‘manufacturing recipe’ concerning compounds other than that requested by the study.

### Considerations: glycerol concentrations in WTS mixture

Concerning regulations of glycerol concentrations in WTS products, it is more reasonable to reference commercial brand samples rather than lab-made samples. Since companies samples are produced by prominent WTS brands in the market, findings from those samples would allow a more direct representation of real-life consumer experience compared to lab-made samples. To confirm declared glycerol concentrations in companies’ samples, validation measurements were conducted by collaborator lab (ISO-accredited Eurofins Food & Feed Testing, Sweden, Lidköping) to eliminate attribution of differences in emissions to differences in glycerol concentrations between compared samples.

Moreover, lab-made samples were prepared as an additional reference to explore how samples prepared from scratch in an experimental lab would compare to market samples. Naturally, WTS mixture contents are not identical in terms of sources, purity grade and overall quality. As an outcome, investigating inconsistencies in findings from lab-made vs. companies’ samples may shed the light on potential confounders affecting the results such as content quality and additive presence.

### Future directions

In order to develop specific regulations for WTS products, there is a need to explore confounders affecting toxicant emissions. Going forward, this may allow identifying enhancement possibilities for WTS product manufacturing. While the focus of this study is glycerol effect on toxicant emissions, a holistic point of view may consider glycerol inhalation toxicity as well for regulatory recommendations regarding waterpipe tobacco content concentrations.

### Strengths and limitations

This study is one of not many studies that have examined levels of glycerol in waterpipe tobacco and the corresponding effect on levels of toxicant emissions. Both brand and generic WTS products were used in this study to compare results, which may lead to further investigations concerning tobacco additives and fillers affecting emissions.

Even though ingredient concentrations in brand tobacco samples were validated by analytical labs for accuracy, other tobacco fillers and additives were not declared by the providing market companies. This alongside the quality of basic ingredients was limiting our conclusion in regards to identifying upper limits of glycerol addition for regulatory recommendations.

## Conclusion

The effect of glycerol addition in waterpipe tobacco on acrolein emission varies between products. Tobacco fillers, additives and contents quality and other factors may affect toxicant emission levels. Therefore, regulatory recommendations towards defining upper limits of content concentrations require further investigations regarding potential confounders in acrolein emissions and health effects of market-available glycerol concentrations in waterpipe tobacco smoking.

## Data Availability

All data generated or analyzed during this study are included in this published article.

## Abbreviations

WTS: Water-pipe tobacco smoking
WHO: Orld Health Organization
SFDA: Saudi Food and Drug Authority
HPLC: High-performance Liquid Chromatography
ISO: International Organization for Standardization
ISO/TS: ISO/Water pipe tobacco smoking
DAD: Diode-Array Detection
LC–MS/MS: Liquid Chromatography—Tandem Mass Spectrometry
eLIMS: Eurofins Laboratory Information Management System
GRAS: Generally Recognized as Safe
LOQ: Limit of Quantification
NAB: N-Nitrosoanabasine
NAT: N-Nitrosoanabatine
NNK: 4-(Methylnitrosamino)-1-(3- pyridyl)-1-butanone
NNN: N-Nitrosonornicotine
